# Mutational landscape of chronic myelomonocytic leukemia in Chinese patients

**DOI:** 10.1186/s40164-022-00284-z

**Published:** 2022-05-24

**Authors:** Yanbo Nie, Liang Shao, Hong Zhang, Colin K. He, Hongyu Li, Junyan Zou, Long Chen, Huaiyue Ji, Hao Tan, Yani Lin, Kun Ru

**Affiliations:** 1 Sino-US Diagnostics Lab, Tianjin Enterprise Key Laboratory of AI-aided Hematopathology Diagnosis, Tianjin, 300385 China; 2 Department of Hematology, Zhongnan Hospital of Wuhan University, Wuhan, 430071 China; 3 Stego Tech LLC, Audubon, PA 19403 USA

**Keywords:** CMML, Gene mutation, Clonal evolution, Secondary AML (sAML), Overall survival (OS)

## Abstract

**Background:**

Chronic myelomonocytic leukemia (CMML) is a rare and heterogeneous hematological malignancy. It has been shown that the molecular abnormalities such as *ASXL1*, *TET2*, *SETBP1*, and *SRSF2* mutations are common in Caucasian population.

**Methods:**

We retrospectively analyzed 178 Chinese CMML patients. The targeted next generation sequencing (NGS) was used to evaluate 114 gene variations, and the prognostic factors for OS were determined by COX regression analysis.

**Results:**

The CMML patients showed a unique mutational spectrum, including *TET2* (36.5%), *NRAS* (31.5%), *ASXL1* (28.7%), *SRSF2* (24.7%), and *RUNX1* (21.9%). Of the 102 patients with clonal analysis, the ancestral events preferentially occurred in *TET2* (18.5%), splicing factors (16.5%), *RAS* (14.0%), and *ASXL1* (7.8%), and the subclonal genes were mainly *ASXL1*, *TET2*, and *RAS*. In addition, the secondary acute myeloid leukemia (sAML) transformed from CMML often had mutations in *DNMT3A, ETV6, FLT3*, and *NPM1*, while the primary AML (pAML) demonstrated more mutations in *CEBPA, DNMT3A, FLT3, IDH1/2, NPM1*, and *WT1*. It was of note that a series of clones were emerged during the progression from CMML to AML, including *DNMT3A, FLT3*, and *NPM1*. By univariate analysis, *ASXL1* mutation, intermediate- and high-risk cytogenetic abnormality, CMML-specific prognostic scoring system (CPSS) stratifications (intermediate-2 and high group), and treatment options (best supportive care) predicted for worse OS. Multivariate analysis revealed a similar outcome.

**Conclusions:**

The common mutations in Chinese CMML patients included epigenetic modifiers (*TET2* and *ASXL1*), signaling transduction pathway components (*NRAS*), and splicing factor (*SRSF2*). The CMML patients with *DNMT3A*, *ETV6*, *FLT3*, and *NPM1* mutations tended to progress to sAML. *ASXL1* mutation and therapeutic modalities were independent prognostic factors for CMML.

**Supplementary Information:**

The online version contains supplementary material available at 10.1186/s40164-022-00284-z.

## Background

Chronic myelomonocytic leukemia (CMML) is a clonal and heterogeneous hematological neoplasm, characterized by persistent monocytosis and hematopoietic dysplasia. Approximately 15–30% of CMML patients progress to secondary acute myeloid leukemia (sAML) [1–5]. The median age of CMML is 65–75 year-old with a disposition to male (1.5–3:1) [6, 7]. According to the WHO classification, CMML is classified into three subtypes: CMML-0 (< 2% in peripheral blood [PB] and < 5% in bone marrow [BM]), CMML-1 (2–4% in PB and 5–9% in BM), and CMML-2 (5–19% in PB and 10–19% in BM). Based on white blood cell (WBC) count, CMML is further defined as myeloproliferative CMML (MP-CMML) or myelodysplastic CMML (MD-CMML) [8].

Recent studies have demonstrated that CMML carried multiple mutations, involving epigenetic regulation, such as DNA methylation (*TET2*, *DNMT3A*, *IDH1/2*) and histone transcription (*RUNX1*, *EZH2*, *ASXL1*), splicing factors (*SF3B1*, *U2AF1*, *ZRSR2*, *SRSF2*), and signaling transduction pathway (*JAK2, KRAS, NRAS, CBL*, *FLT3*) [7, 9–12]. Among them, mutations in *TET2*, *SRSF2*, and *ASXL1* tended to occurred in MD-CMML, whereas mutations in *RAS* signaling transduction pathway were more prevalent in MP-CMML [13, 14]. The prognostic system for CMML generally included clinical features and lab findings, such as blood/bone marrow biopsies, as well as mutations in *ASXL1*, *NRAS*, *RUNX1*, and *SETBP1* et al. [15–20].

To reveal the molecular landscape, we retrospectively analyzed 178 Chinese CMML patients and investigated their mutational spectrum. The clonal dynamics upon disease progression from CMML to sAML was studied, and the prognostic factors for the overall survival (OS) of CMML patients were evaluated.

## Methods

### Patients

A total of 411patients from June 2015 to January 2021 were collected and sorted in this study, including 178 CMML, 13 sAML (3 transformed from the CMML cases), and 223 primary AML (AML-M4/M5). The risk stratification was as follows [1, 2]: low-risk, a diploid karyotype or sole -Y; high-risk, trisomy 8, alterations of chromosome 7, or complex karyotype; intermediate-risk, all other karyotypes. In addition, 92 CMML patients with available clinical information were evaluated for OS in our cohort. The median follow-up time was 32 months. The OS was calculated from the day of diagnosis to the day of death regardless of cause or last contact. This study was approved by the IRB of Sino-US Diagnostics.

### Next-generation sequencing (NGS)

A total of 114 genes associated with hematological disorders were analyzed using NGS (Additional file 1: Table S1). Genomic DNAs were extracted from the BM mononuclear cells according to the manufacturer’s instructions (TIANGEN, China). The genes were amplified in the 275-bp libraries by customized primers (Life Technologies, USA), and the primers covered the entire coding regions and canonical splice sites. The amplified products were sequenced on Ion Torrent platform (Life Technologies, USA). The average sequencing depth was 1000× per patient and mutations with VAF (variant allele frequency) > 2% were considered positive.

The variants were annotated by ANNOVAR using following resources: RefGene, public population databases, 1000 Genomes Project Phase 3 database, protein function prediction databases [21, 22], and in-house database. These databases were listed in Additional file 2: Table S2. In addition, some variants, such as frameshift mutations or total reads less than 30, were manually reviewed using the Integrative Genomics Viewer (Broad Institute, bam) [23].

### Ancestral vs. sub-clonal variants

To distinguish the ancestral/founder from the secondary/subclonal variants, the VAF was used to estimate the clonal hierarchy of each sample and the following criteria were formulated: (a) a cut-off value of at least 5% difference between VAFs was used to define an ancestral mutation. If the difference was less than 5%, a co-dominant was called [24–26]; and (b) to analyze the transformed cases, mutations appearing at progression to CMML-2/AML but not present initially were deemed subclones [27]. The ambiguous data were disregarded.

### Statistical analysis

The Chi-square test was used to compare the enumeration data between different groups, and the student t-test or analysis of variance was used to compare the measurement data. All statistical analyses were performed using SPSS version 24.0 (IBM, Armonk, NY, USA) and R software. Correlations between mutations were conducted by Pearson coefficients. The forest plots were performed by the GraphPad Prism 7.0. The univariate analysis was performed to reveal the OS related factors, and the multivariate Cox proportional hazards regression analysis was used to further evaluate the factors with p < 0.05 from the univariate analysis. The OS curve was plotted by the Kaplan-Meier method and tested by the log-rank. All p-values were two-tailed, and p < 0.05 was considered to be statistically significant.

## Results

### Clinical characteristics

The study included 178 CMML patients with 65 CMML-0, 58 CMML-1, and 55 CMML-2. The median age was 62 year-old (17–90), and 66.3% of CMML patients were male. Most of MD-CMML patients (66/83; 79.5%) presented in the low-risk group, more than MP-CMML patients (63/95; 66.3%). The MP-CMML patients preferentially exhibited splenomegaly than the MD-CMML patients (p = 0.036). Moreover, the MP-CMML patients showed significantly higher levels of absolute neutrophil count (ANC), absolute monocyte count (AMC), and lactate dehydrogenase (LDH) than the MD-CMML patients (p = 0.000, p = 0.002, p = 0.008, respectively) (Table 1). The CMML-0 had more low-risk patients (55/65; 84.6%) in contrast to the CMML-1 (38/58, 65.5%; p = 0.014) and CMML-2 (36/55, 65.5%; p = 0.015), whereas the CMML-1 (13/58, 22.4%; p = 0.021) and CMML-2 (13/55; 23.6%; p = 0.015) tended to be in the high-risk group compared with the CMML-0 (5/65, 7.7%). Up to 28.4% of CMML patients exhibited splenomegaly. The platelet (PLT) count increased along with the higher disease stage (p = 0.015), whereas the WBC count showed a reverse trend (p = 0.007). Most  CMML-1/2 patients received hypomethylating agents (HMAs) ± chemotherapy/allogeneic hematopoietic stem cell transplantation (allo-HSCT) (Table 2).

**Table 1:** The clinical information of CMML patients classified as MD/MP subtypes

Variables	Total CMML (n = 178)	MD-CMML (n = 83)	MP-CMML (n = 95)	*P* value
Age in years; median (range)	62 (17–90)	63 (24–90)	60 (17–85)	0.362
Sex (male); n (%)	118 (66.3%)	54 (65.1%)	64 (67.4%)	0.745
Hemoglobin g/L; median (range)	85 (38–173)	85 (38–140)	87 (40–173)	0.410
ANC ×10^9^/L; median (range)	4.75 (0.45–29.97)	2.17 (0.45–6.15)	10.9 (1.14–29.97)	0.000
AMC ×10^9^/L; median (range)	2.43 (1.01–16.32)	1.8 (1.01–5.69)	3.23 (1.11–16.32)	0.002
Platelets×10^9^/L; median (range)	76 (3–977)	69 (11–900)	85 (3–977)	0.828
LDH IU/ml; median (range)	279.1 (120–1403)	224.6 (120–977.1)	333.5 (152.4–1403)	0.008
Cytogenetic risk group^a^ n (%)				0.032
Low-risk	129 (72.5%)	66 (79.5%)	63 (66.3%)	0.049
Intermediate-risk	18 (10.1%)	9 (10.8%)	9 (9.5%)	0.762
High-risk	31 (17.4%)	8 (9.7%)	23 (24.2%)	0.011
Splenomegaly ; n (%)	42 (28.4%)	13 (19.6%)	29 (35.3%)	0.036
Treatment strategy; n (%)				0.821
Best supportive care	28 (30.4%)	12 (28.6%)	16 (32.0%)	
HMAs ± chemotherapy/allo-HSCT	64 (69.6%)	30 (71.4%)	34 (68.0%)	

FAB classification MD-CMML: WBC < 13 × 109/L,  (109: 9 needs to be superscripted) MP-CMML: WBC ≥ 13 × 109/L  (109: 9 needs to be superscripted)

LDH: Lactate dehydrogenase

^a^Cytogenetic risk groups [1, 2]: low-risk: a diploid karyotype or -Y; high-risk: trisomy 8, alterations of chromosome 7, as well as complex karyotype; intermediate-risk: all other karyotypes

**Table 2:** The clinical information of 178 CMML patients classified as CMML-0, -1, and -2

Variables	CMML-0 (n = 65)	CMML-1 (n = 58)	CMML-2 (n = 55)	*P* value	0 vs. 1	0 vs. 2	1 vs. 2
Age in years; median (range)	65 (24–86)	62 (23–82)	59 (17–90)	0.085	–	–	–
Sex (male); n (%)	41 (63.1%)	43 (73.1%)	34 (61.8%)	0.383	–	–	–
Hemoglobin (g/L); median (range)	89 (53–173)	82 (38–153)	84.0 (39–131)	0.049	0.267	0.058	1.000
WBC (×10^9^/L); median (range)	9.32 (1.38–158.9)	12.79 (3.15–177.7)	23.7 (1.22-323.66)	0.007	1.000	0.007	0.068
ANC (×10^9^/L); median (range)	4.66 (0.51–32)	3.92 (0.45–20.62)	6.35 (1.06–29.97)	0.606	–	–	–
AMC (×10^9^/L); median (range)	2.4 (1.11–6.5)	2.43 (1.01–6.45)	2.85 (1.01–16.32)	0.600	–	–	–
Platelets(×10^9^/L); median (range)	101 (11–977)	66 (6-900)	62 (3-610)	0.015	0.046	0.037	1.000
LDH (IU/ml); median (range)	230.5 (120–1403)	266 (152.4–623)	352 (121.6–1298)	0.046	1.000	0.097	0.136
Cytogenetic risk group^a^; n (%)							
Low-risk	55 (84.6%)	38 (65.5%)	36 (65.5%)	0.023	0.014	0.015	0.994
Intermediate-risk	5 (7.7%)	7 (12.1%)	6 (10.9%)	0.704	–	–	–
High-risk	5 (7.7%)	13 (22.4%)	13 (23.6%)	0.034	0.021	0.015	0.877
Splenomegaly; n (%)	16 (28.6%)	12 (26.1%)	14 (30.4%)	0.898	–	–	–
Treatment strategy; n (%)							
Best supportive care	17 (51.5%)	6 (23.1%)	5 (15.2%)	0.004	0.034	0.004	0.512
HMAs ± chemotherapy/allo-HSCT	16 (48.5%)	20 (76.9%)	28 (84.8%)				

(WHO CMML-0: PB < 2%, BM < 5%; CMML-1: PB = 2–4%, BM = 5–9%; CMML-2: PB = 5–9%, BM = 10–19%)

^a^Cytogenetic risk groups [1, 2]: low-risk: a diploid karyotype or -Y; high-risk: trisomy 8, alterations of chromosome 7, as well as complex karyotype; intermediate-risk: all other karyotypes

### The spectrum of gene mutations

**Fig. 1:**
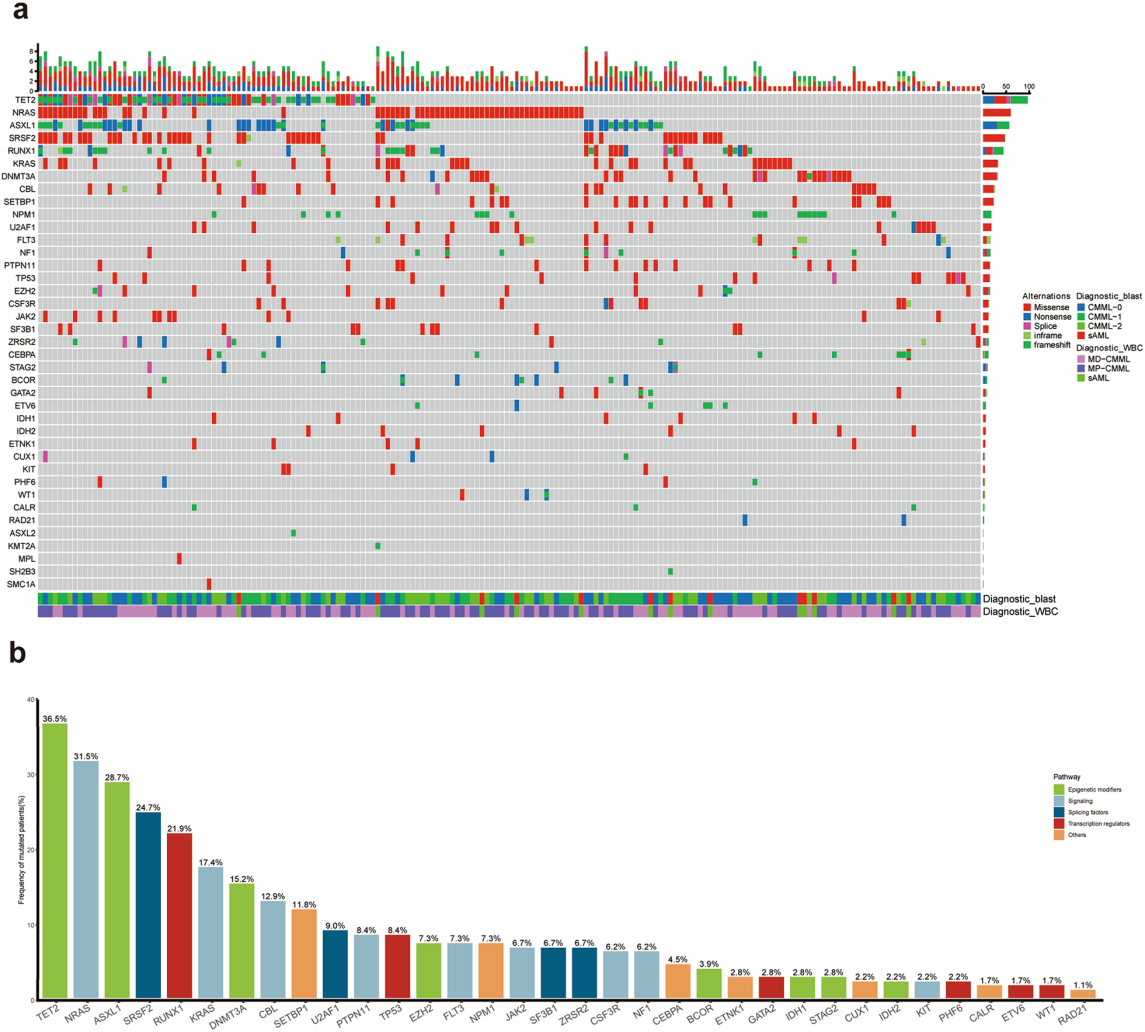
**a** The distribution of somatic mutations in CMML. Each column represents a patient and each row corresponds to a gene. The color of each rectangle represents the type of gene mutation, the diagnosis, and the karyotype of each patient. The bar graph indicates the mutation frequency, the mutation type, and the cytogenetics of each patient; **b** The overview of pathways involving in the identified mutations. The histogram represents the frequency of common gene mutations, and the mutation frequency is expressed as a percentage. *FLT3* mutations consist of *FLT3-*ITD and *FLT3-*TKD

The targeted panel NGS was performed in 178 CMML, 13 sAML (3 transformed from CMML), and 223 pAML. As shown in Fig. 1a, the CMML patients had a unique mutational spectrum, and 83.1% (148/178) carried at least two oncogenic mutations. The common mutations included *TET2* (36.5%), *NRAS* (31.5%), *ASXL1* (28.7%), *SRSF2* (24.7%), and *RUNX1* (21.9%) (Fig. 1b). Among those mutations, *TET2* and *RUNX1* had multiple mutation forms, including frameshift/inframe, nonsense, splicing, and missense mutations. *ASXL1* preferentially presented with nonsense or frameshift mutations, whereas *N/KRAS*, *SRSF2*, *SETBP1*, *DNMT3A*, and *CBL* were mainly missense mutations.

**Fig. 2:**
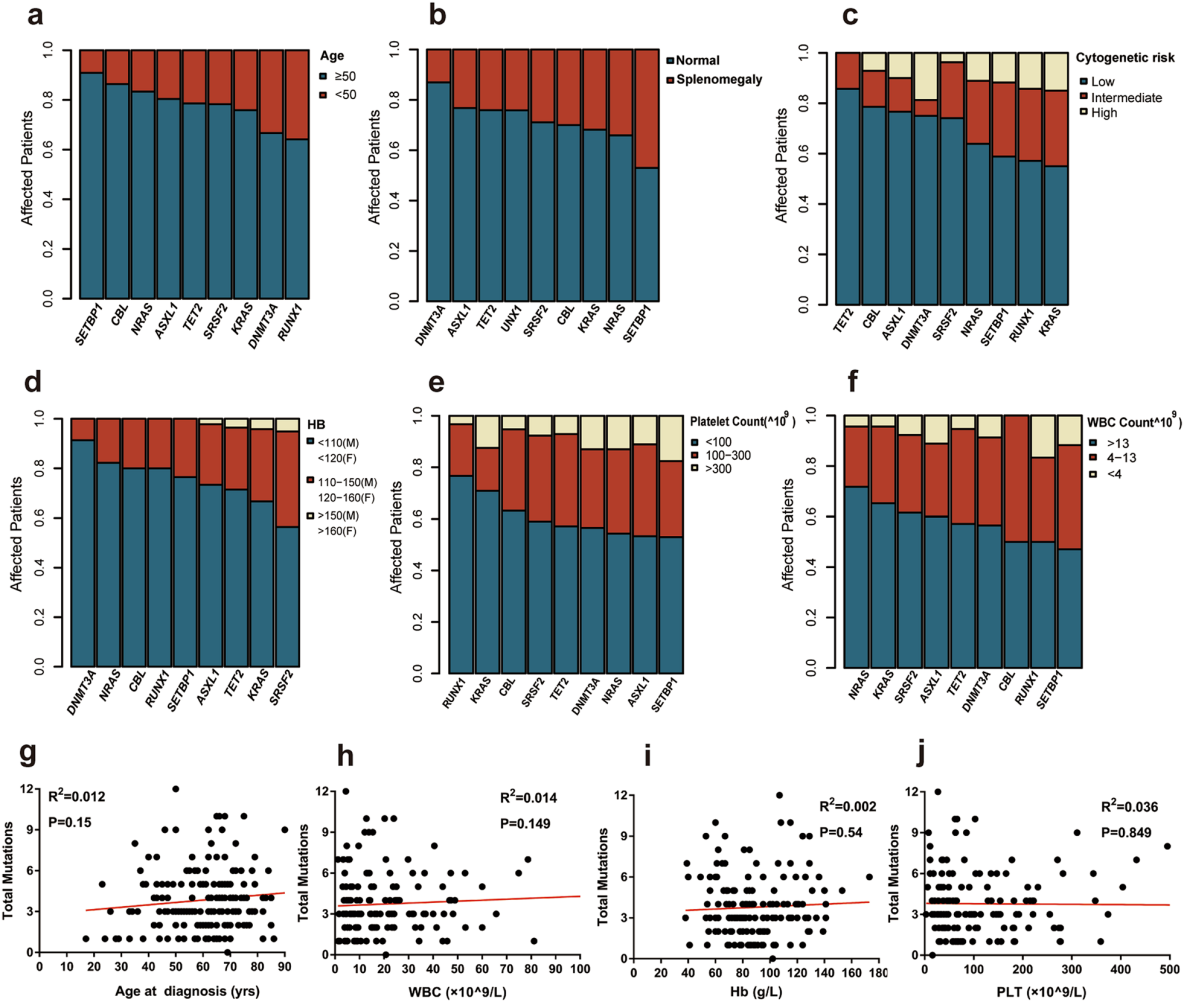
The proportions of patients with various mutations based on age (**a**), splenomegaly (**b**), cytogenetic risk (**c**), HB (**d**), PLT (**e**), and WBC (**f**). The correlation of total CMML mutations versus age at diagnosis (**g**), WBC (**h**), Hb (**i**), and PLT level (**j**)

Further analysis showed that *RUNX1* mutation occurred preferentially in CMML patients less than 50 year-old (p < 0.05). Additionally, patients with *SRSF2* mutation were prone to have normal hemoglobin (Hb) level (p < 0.05), whereas patients with *RUNX1* mutation tended to have lower PLT count (p = 0.05). Notably, *NRAS* mutation was correlated with leukocytosis (p < 0.05) (Fig. 2a–f). No significant correlation was found between the total number of mutations and age, Hb, PLT count, or WBC count (Fig. 2g–j).

### The correlation between genetic mutations

**Fig. 3:**
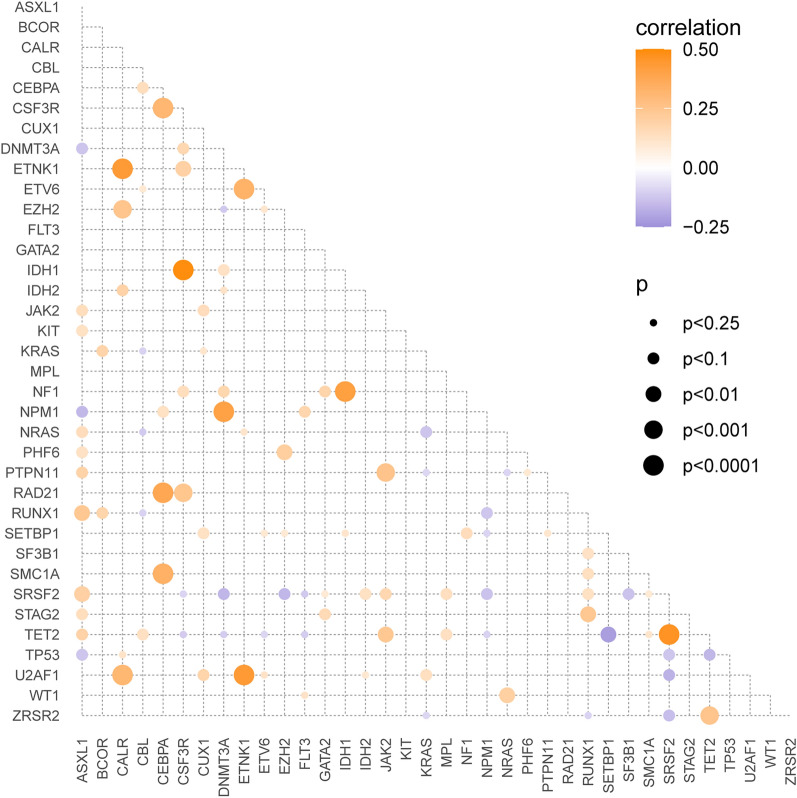
The relationships among the driver mutations in CMML patients. The red and blue circles represent co-occurrence and mutually exclusive changes respectively. The circle size indicates the size of the effect, and the p value is expressed by colored gradient

As shown in Fig. 3, a co-mutation was detected between *IDH1* and *CSF3R* or *NF1* (r = 0.490, p < 0.0001; r = 0.414, p < 0.0001, respectively). Similarly, *SRSF2* mutation co-presented with *TET2* mutation (r = 0.465, p < 0.0001), so did *ETNK1* and *U2AF1, CALR*, or *ETV6* mutations (r = 0.439, p < 0.0001; r = 0.435, p < 0.0001; r = 0.331, p < 0.001, respectively), as well as *DNMT3A* and *NPM1* mutations (r = 0.404, p < 0.0001). By contrast, *TET2* and *SETBP1* mutations were mutually exclusive (r=-0.219, p < 0.01).

### Ancestral and sub-clonal events

**Fig. 4:**
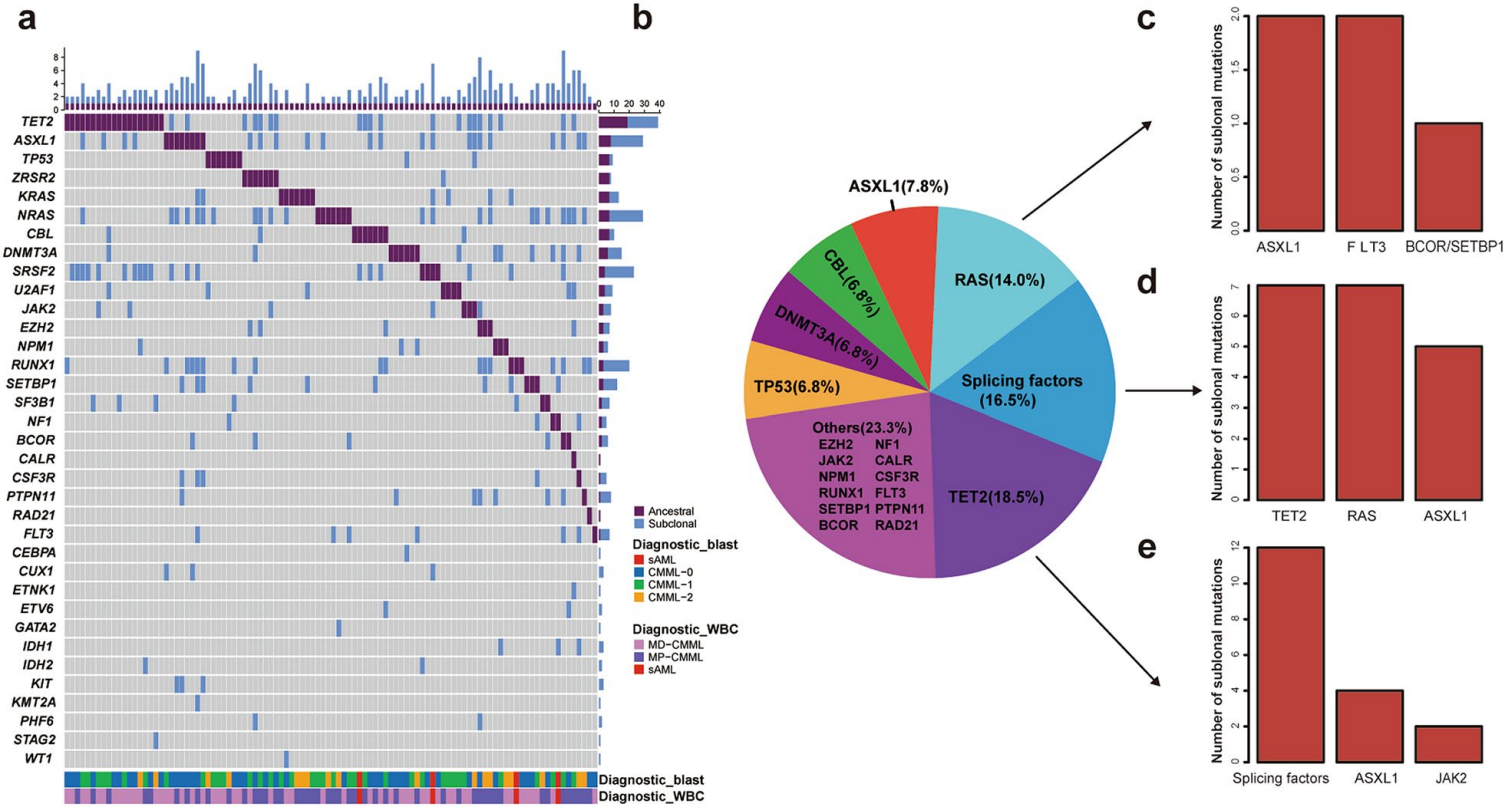
The ancestral and subclonal events in CMML patients. **a** The distribution of the ancestral and subclonal mutations. Each column represents a patient and each row corresponds to a gene. The bar graph on the right represents the number of primary clones or subclones of each gene. A purple square represents one ancestral clonal event, and a blue square represents one subclonal event. **b** The pie chart lists the distribution of more than 6% of ancestral genes. **c**–**e** In CMML, the bar graphs represent the most common subclonal events for the first three ancestral events

To analyze the ancestral and subclonal events in the 178 CMML patients, mutations with highest VAF were defined as the ancestral/dominant mutation, and others with similar VAFs were defined as co-dominant. As shown in Figs. 4a and 57.3% (102/178) of the patients had a single unique dominant event. Of the 102 patients, the ancestral events preferentially occurred in the following genes: *TET2* (18.5%), splicing factors (16.5%), *RAS* (14.0%), *ASXL1*(7.8%), *CBL* (6.8%), *DNMT3A* (6.8%), and *TP53* (6.8%); the subclonal genes were mainly *ASXL1*, *TET2*, and *NRAS*. (Fig. 4a, b). The ancestral events with *TET2* mutations were concomitantly accompanied by splicing factors, *ASXL1*, and *JAK2* subclones, whereas the ancestral events with splicing factors mutations were accompanied by *TET2*, *RAS*, and *ASXL1* subclones. The ancestral events with *RAS* signaling mutations were usually accompanied by *ASXL1* and *FLT3* subclones (Fig. 4c–e).

### Driver mutation enrichment in CMML and sAML

**Fig. 5:**
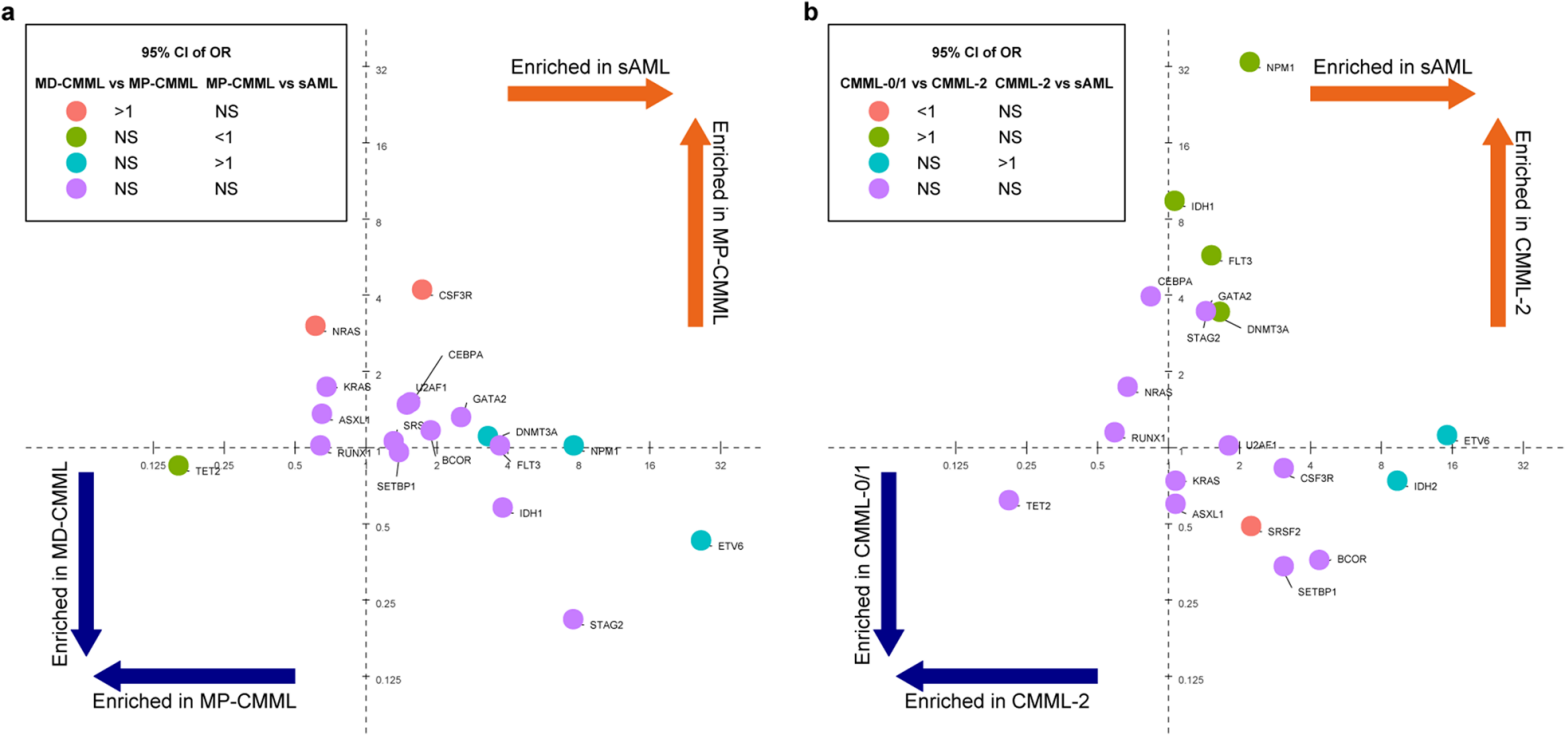
The driver mutations from CMML subtypes and sAML. **a** The enrichment shows the odds ratio (OR) of mutation rates in sAML (n = 13) vs. MP-CMML (n = 95) and MP-CMML (n = 95) vs. MD-CMML (n = 83) on the x-axis and y-axis, respectively. **b** The enrichment shows the OR of mutation rates in sAML (n = 13) vs. CMML-2 (n = 55) and CMML-2 (n = 55) vs. CMML-0/1 (n = 123) on the x-axis and y-axis, respectively The enrichments from the comparisons are indicated by colors according to odds ratios (OR) and 95% confidence intervals (CI) limits being above (if OR > 1) or below (if OR < 1)

The enrichment of driver mutations in various malignancies has been observed. As seen in Fig. 5a, mutations in *NPM1*, *ETV6, FLT3*, and *DNMT3A* were preferentially enriched in sAML, *TET2* mutation was enriched in MD-CMML, and *NRAS* mutation was enriched in MP-CMML. It was also found that *IDH1, FLT3, NPM1, DNMT3A*, and *ETV6* mutations were enriched in CMML-2 and/or sAML, and *TET2* mutation was enriched in CMML-0/1 using univariate comparison (Fig. 5b). Those results indicated that *ETV6*, *FLT3*, *DNMT3A*, and *NPM1* mutations may play a role in the transformation from CMML to sAML.

### Prevalent gene mutations in CMML and pAML

**Fig. 6:**
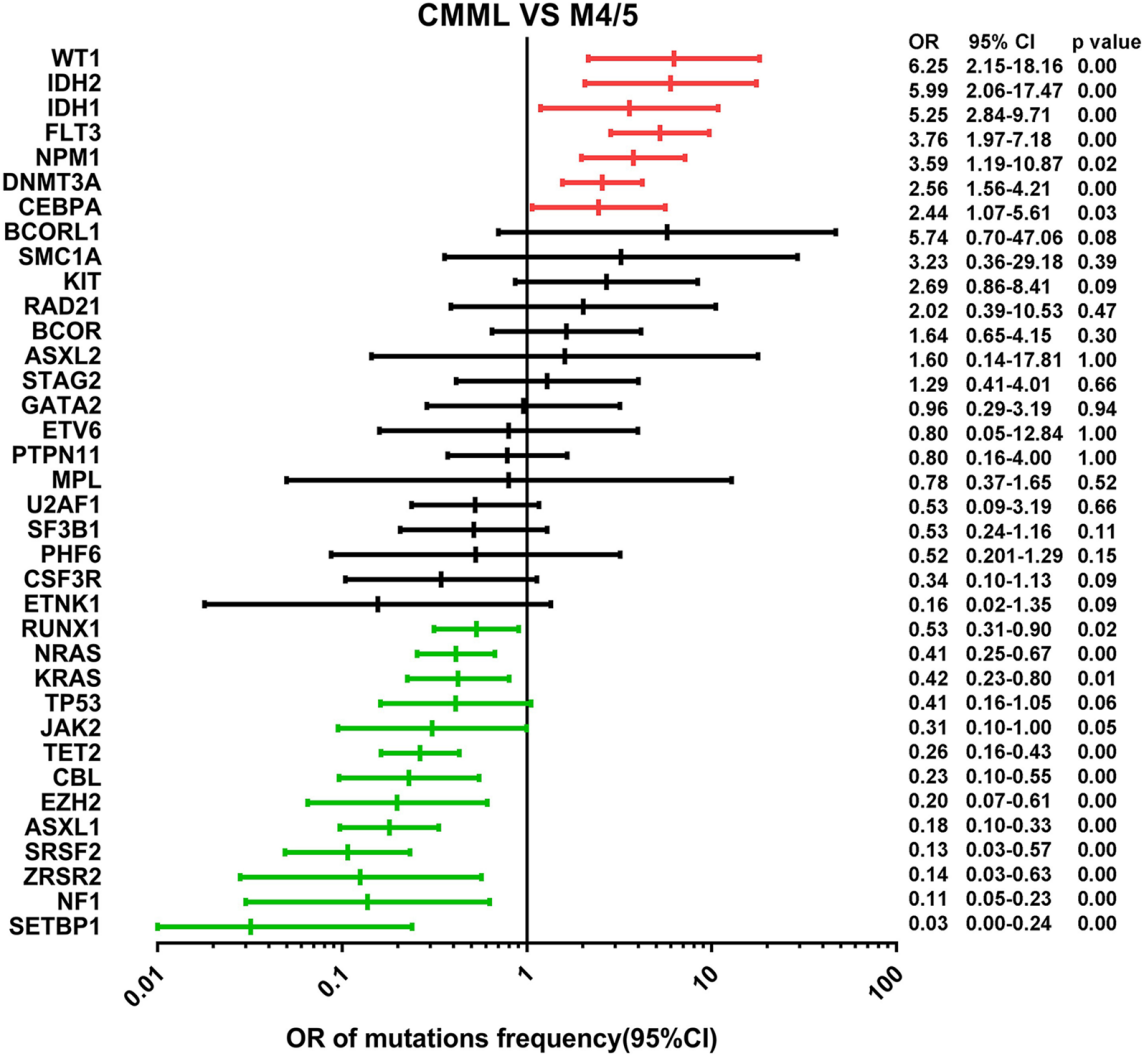
The forest plot with the OR and 95% CI of rates from the common driver mutations between CMML and pAML. The significant discrimination is shown in red and green lines

Two different mutational spectrums were found when CMML and pAML were co-evaluated. As shown in Fig. 6, seven mutations including *WT1* (OR 6.25; p = 0.00), *IDH2* (OR 5.99; p = 0.00), *IDH1* (OR 5.25; p = 0.00), *FLT3* (OR 3.76; p = 0.00), *NPM1* (OR 3.59; p = 0.02), *DNMT3A* (OR 2.56; p = 0.00), and *CEBPA* (OR 2.44; p = 0.03) were enriched in pAML, whereas twelve mutations from *RUNX1* (OR 0.53; p = 0.02), *NRAS* (OR 0.41; p = 0.00), *KRAS* (OR 0.42; p = 0.01), *JAK2* (OR 0.31; p = 0.05), *TET2* (OR 0.26; p = 0.00), *CBL* (OR 0.23; p = 0.00), *EZH2* (OR 0.20; p = 0.00), *ASXL1* (OR 0.18; p = 0.00), *SRSF2* (OR 0.13; p = 0.00), *ZRSR2* (OR 0.14; p = 0.00), *NF1* (OR 0.11; p = 0.00), and *SETBP1* (OR 0.03; p = 0.00) were enriched in CMML.

### Clonal dynamics

**Fig. 7:**
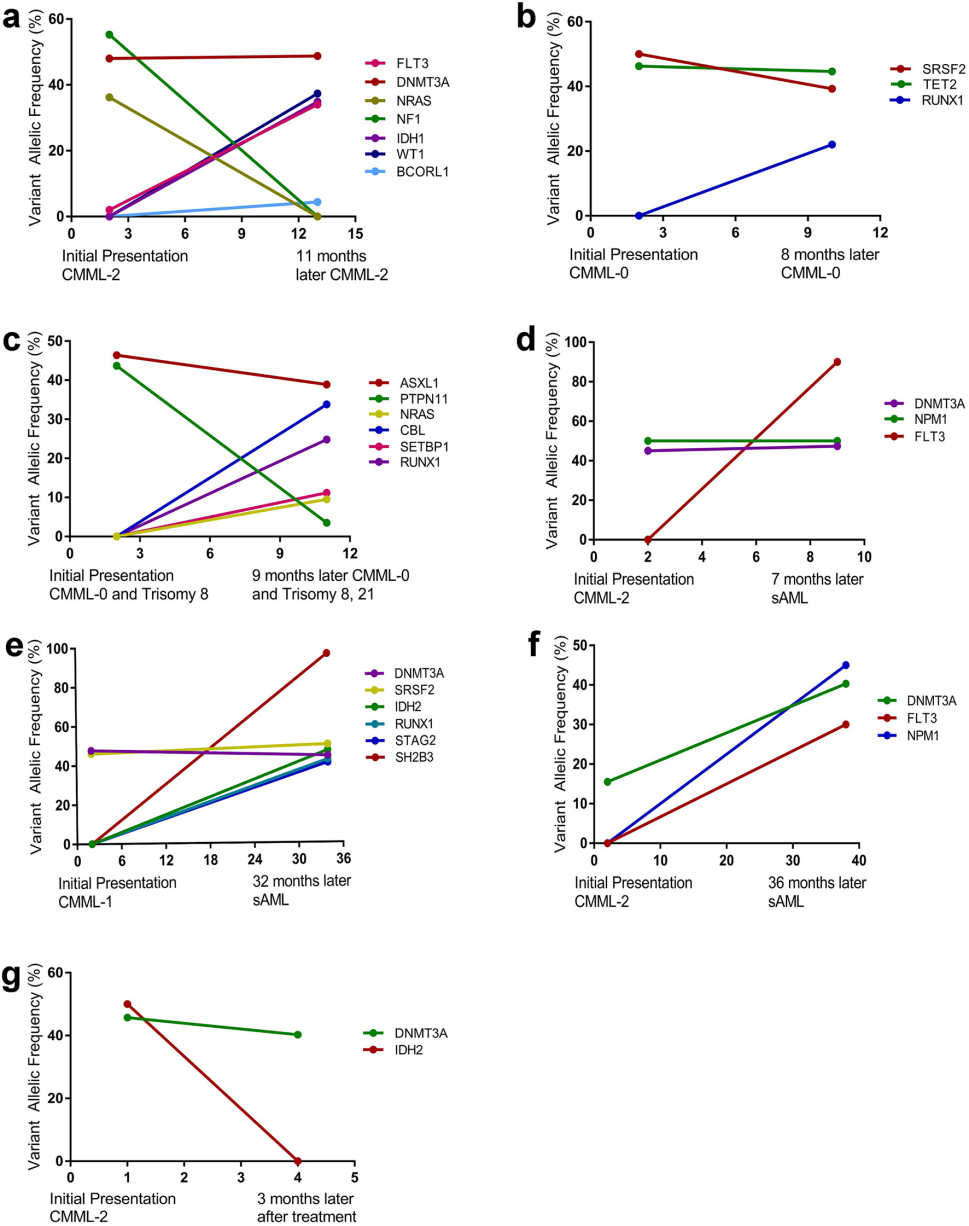
The clonal evolution in CMML. The clonal dynamics from seven patients with the VAF of numerous mutations (**a**–**g**) using different line colors, followed by the acquisition of new mutations (**a**–**f**), cytogenetic abnormalities (**c**), and progression (**d**–**f**)

Clonal evolution was a common phenomenon in cancers, especially during disease progression, so as to CMML. The clonal dynamics from seven patients were evaluated with available molecular data, including 2 cases of CMML-0, 2 cases of CMML-2, and 3 cases of sAML transformed from CMML. The secondary clones were divided into emerging and vanishing clones. As demonstrated in Fig. 7, the emerging/increasing clones were *FLT3/WT1* (Fig. 7a), *RUNX1* (Fig. 7b), *CBL/ RUNX1/SETBP1/NRAS* (Fig. 7c), *FLT3* (Fig. 7d), *SH2B3/IDH2/STAG2/RUNX1* (Fig. 7e), and *DNMT3A*/*FLT3*/*NPM1* (Fig. 7f). The vanishing/decreasing clones included *NF1*/*NRAS* (Fig. 7a), *SRSF2* (Fig. 7b), *PTPN11*/*ASXL1* (Fig. 7c), and *DNMT3A/IDH2* (Fig. 7g). An illustrative example was shown in Fig. 7d–f, including three sAML cases transformed from CMML. Among them one acquired an emerging *FLT3* mutation, another one acquired three emerging mutations (*FLT3, DNMT3A*, and *NPM1*), and the third one acquired an emerging *SH2B3* mutation.

### Survival analysis

**Fig. 8:**
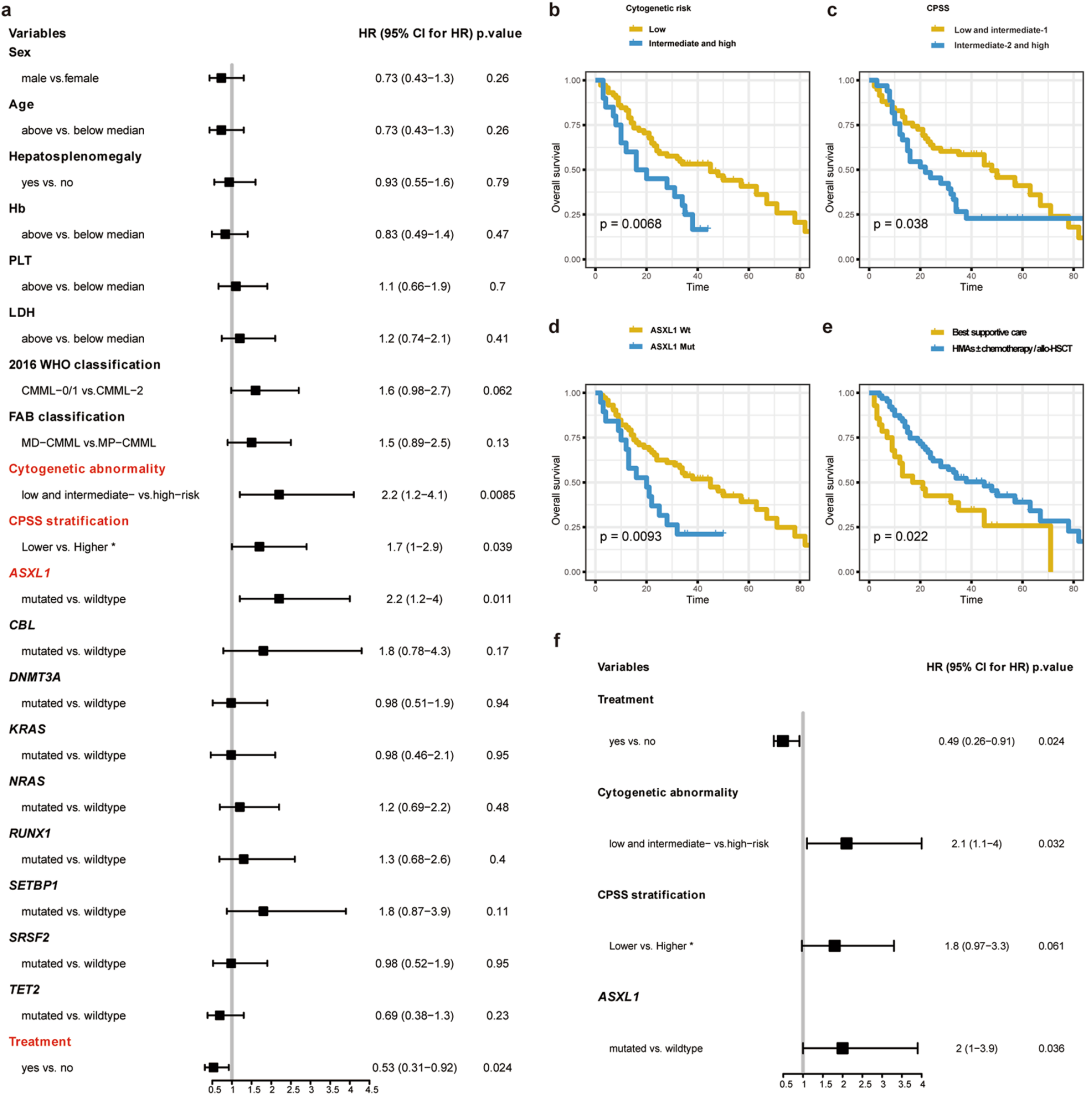
The univariate and multivariate Cox regression analysis of clinical and biological factors influencing OS. **a** The univariate analysis defines these variables by the hazard ratio (HR) with 95% CI, and gene mutations are included in the analysis if presenting in at least 10% patients. **b** Survival curves are charted for variables affecting the OS. **c** The multivariate analysis contains the index if presenting in the univariate analysis with p-value < 0.05 for OS

The univariate analysis included gender, adjusted age, splenomegaly, Hb, PLT, LDH, 2016 WHO classification, FAB subtypes, cytogenetic abnormality, CPSS risk stratifications, gene mutations and total number, and treatment options. Among them, cytogenetic abnormality [intermediate- and high-risk, hazard ratio (HR): 2.2, 95% confidence intervals (CI) 1.2–4.1; p = 0.009], and CPSS risk stratifications (intermediate-2 and high group, HR: 1.7, 95% CI 1.0-2.9; p = 0.039), and *ASXL1* mutation [HR: 2.2, 95% CI 1.2-4.0; p = 0.011] indicated a shorter OS, and treatment modalities (HMAs ± chemotherapy/allo-SCT) (HR: 0.53, 95% CI 0.31–0.92; p = 0.024) predicted a better OS (Fig. 8a–e). The multivariate analysis revealed a similar result, except that CPSS risk stratifications (intermediate-2 and high group, HR: 1.8, 95% CI 0.97–3.3; p = 0.061) was not associated with the prognosis (Fig. 8f).

## Discussion

In this study 178 CMML cases were analyzed including demographic, clinical, and laboratory features. This has been the largest cohort of CMML in Chinese population so far. Our study revealed multiple mutations in CMML and investigated their correlation during the clonal evolution. The paired data of gene mutations in CMML between Chinese and Caucasian patients were as follows: *TET2* (36.5% vs. 29–61%), *ASXL1* (28.7% vs. 30–50%), *SRSF2* (24.7% vs. 29–52%), *KRAS* (17.4% vs. 7–16%), *CBL* (12.9% vs. 8–22%), *RUNX1* (21.9% vs. 8–25%), and *SETBP1* (11.8% vs. 4–18%). Nevertheless, Chinese patients gained higher mutation rates in *NRAS* (31.5% vs. 4–25%), *DNMT3A* (15.2% vs. 2–12%), *PTPN11* (8.4% vs. < 5%), *TP53* (8.4% vs. 1–3%), *FLT3* (7.3% vs. 1–3%), and *NPM1* (7.3% vs. 1–3%) [9, 14]. It has been reported that mutations in *RAS/MAPK* signaling genes such as *NRAS* and *PTPN1*1 were associated with CMML-MP [1, 3, 28]. Both *NRAS* and *DNMT3A* mutations played a role in CMML transformation to AML [29, 30]. *NRAS* mutations were usually obtained in the later stage of clonal hematopoiesis, and transplanted CMML patients with *NRAS* mutation had an unfavorable prognosis [28]. Azacitidine and trametinib delivered a synergistic effect in *NRAS*-mutated CMML [31]. Several studies demonstrated that *DNMT3A* mutation was an independent adverse prognostic factor for OS and LFS [12, 32, 33]. CMML patients with *NPM1* and *FLT3* mutations tended to have a rapid progress to AML [3, 9, 28, 34], so did *TP53* mutation [3, 35, 36]. The similar findings in our cohort suggested that those genes support a clonal expansion in CMML, as well as leading to a poor prognosis.

The order of acquired molecular abnormalities is particularly important. The ancestral/primary clonal lesions usually present the main clinical phenotypes, and the secondary or tertiary mutations likely predict disease progression. Several studies identified that *TET2* and *ASXL1* were the most predominant primary/ancestral events in CMML patients [24, 27, 37]. Unlike the reported studies, our investigation demonstrated that splicing factors (*SRSF2*, *U2AF1*, *SF3B1*, and *ZRSR2*) and *RAS* related genes (*NRAS* and *KRAS*) were common in primary/ancestral events. *ASXL1* and *FLT3* mutations were considered to be the adverse prognostic factors in CPSS-mol and MMM prognostic stratification systems [6, 9, 20]. In addition, the ancestral and subclonal events are exchangeable. The driver genes such as *IDH1/CSF3R, IDH1/NF1, TET2/SRSF2* [2, 10, 28, 38, 39] and *ETNK1/U2AF1* could mutate simultaneously, resulting in an instability of the genome and promoting the acquisition of additional mutations. On the other side, mutations in *TET2/SETBP1* was observed in a mutually exclusive manner [33, 40].

During the transformation of CMML to sAML, one distinct feature was the emergence of new gene mutations or increase of VAF [41]. It was of note that a series of clones were emerged or increased from the seven transformed CMML cases in our study, including *FLT3*, *NPM1*, *IDH2*, *DNMT3A* et al. Some newly acquiring mutations, the so-called type 1 mutations *FLT3*, *NPM1,* and *IDH2*, seemed to predict a much shorter time for disease progression from MDS to sAML than type 2 mutations that were present in MDS [41–44]. The mutational tendency was evident in our study, and mutations in *WT1*, *IDH2*, *IDH1*, *FLT3*, *NPM1*, *DNMT3A,* and *CEBPA* were more common in pAML than in CMML. In addition, sAML and pAML overlapped with *FLT3*, *NPM1*, *DNMT3A* mutations, while *WT1* and *IDH1/2* mutations typically occurred in pAML. Therefore, closely monitoring of the emergence of *DNMT3A*, *ETV6*, *FLT3,* and *NPM1* mutations would be an efficient way to predict the early transformation of CMML.

*ASXL1* mutation (only nonsense and frameshift mutations) was an independent prognostic factor in our study by univariate and multivariate analyses, which was consistent with Mayo molecular model (MMM) and CPSS molecular model (CPSS-mol) [1, 6, 45]. The treatment options for CMML were various given the fact of disease heterogeneity. The strategies included: best supportive treatment, HMAs, chemotherapy, and allo-HSCT [46, 47]. In our study, the Cox model showed that treatment modalities (HMAs ± chemotherapy/allo-HSCT) was closely correlated with a better OS. A retrospective study showed that HMAs monotherapy group had a prolonged OS and achieved high response, but could not significantly modify the disease process [48]. In addition, the universal applicability of this treatment option was limited by the complication of allo-HSCT, such as non-recurrent mortality (NRM) and acute and chronic graft-versus-host disease (GVHD) [5, 47]. An open-label, non-randomized phase 2 clinical trial is undergoing to evaluate the efficacy of cobimetinib, a selective a reversible ATP-noncompetitive MEK inhibitor, in CMML patients with activated *RAS* pathway (mutations in *NRAS*, *KRAS*, *PTPN11*, *FLT3*,*CBL*, *JAK2*, *BRAF,* and *NF1* at VAF ≥ 5%) (NCT04409639) [49]. The gain of function mutation in *SRSF2* resulted in transcriptome-wide mis-splicing, leaving the mutated cells more susceptible to splicing inhibitory molecules than their counterpart wild-type cells [50, 51]. H3B-8800, a splicing modulator, is currently being investigated in patients with MDS, AML, and CMML (NCT02841540). These data suggested that specific molecular targets for gene mutations be an alternative and effective choice for the treatment of CMML.

## Conclusions

The molecular profile of Chinese CMML patients displayed a wide range of mutations, and the emerging genes such as *DNMT3A*, *ETV6*, *FLT3,* and *NPM1* usually indicated a dismal outcome of disease progression. In addition to the cytogenetic abnormality and treatment options (best supportive care), *ASXL1* mutation also negatively affected the OS. Therefore, those molecules provided some potential biomarkers for diagnostic and prognostic prediction.

## Supplementary Information

**Additional file 1**: **Table S1.** Thelist of 114 genes by NGS.

**Additional file 2**: **Table S2.** Reference database website.

## Data Availability

The data generated and analyzed in the current study are not public, but it can be obtained from the authors upon request.

## Abbreviations

CMML: Chronic myelomonocytic leukemia
sAML: Secondary acute myeloid leukemia
pAML: Primary AML
MDS: Myelodysplastic syndrome
PB: Peripheral blood
BM: Bone marrow
WHO: World Health Organization
MP-CMML: Myeloproliferative CMML
MD-CMML: Myelodysplastic CMML
WBC: White blood cell
Hb: Hemoglobin
PLT: Platelet
WES: Whole-exome sequencing
VAF: Variant allelic frequency
NGS: Next-generation sequencing
ExAc: Exome aggregation consortium
ESP6500: Exome sequencing project 6500
OR: Odds ratio
HR: Hazard ratio
CI: Confidence intervals
OS: Overall survival
