# Supplementary material for: Mutational landscape of chronic myelomonocytic leukemia in Chinese patients
Source: Exp Hematol Oncol. 2022 May 24;11:32. doi: 10.1186/s40164-022-00284-z (PMC9128105; doi:10.1186/s40164-022-00284-z)
Supplement: Supplementary file 1 — Additional file 1: Table S1. Thelist of 114 genes by NGS. [file 40164_2022_284_MOESM1_ESM.docx]

**Additional file 1: Table S1.** The list of 114 genes by NGS.

| **Gene** | **Region** | **Gene** | **Region** | **Gene** | **Region** |
| --- | --- | --- | --- | --- | --- |
| ABL1 | CDS | CD28 | Exon4 | ETV6 | CDS |
| ANKRD26 | Exon1/5’UTR | CD58 | Exon2-3 | EZH2 | CDS |
| ARID1A | CDS | CD79B | CDS | FAM46C | CDS |
| ASXL1 | CDS | CDC25C | Exon8 | FAT1 | CDS |
| ASXL2 | CDS | CDKN1B | CDS | FBXW7 | CDS |
| ATG2B | CDS | CDKN2A | CDS | FGFR3 | CDS |
| ATM | CDS | CEBPA | CDS | FLT3 | CDS |
| B2M | CDS | CNOT3 | Exon2-5 | GATA1 | CDS |
| BCL2 | Exon2 | CREBBP | CDS | GATA2 | CDS |
| BCL6 | 5’UTR | CRLF2 | Exon6 | GATA3 | CDS |
| BCOR | CDS | CSF3R | CDS | GNA13 | Exon1-4 |
| BCORL1 | CDS | CSNK1A1 | Exon2-4 | ID3 | CDS |
| BIRC3 | CDS | CUX1 | CDS | IDH1 | CDS |
| BRAF | CDS | CXCR4 | CDS | IDH2 | CDS |
| BRINP3 | CDS | DDX3X | CDS | IKZF1 | CDS |
| BTK | Exon5/11/14-19 | DDX41 | CDS | IL7R | Exon5-6 |
| CALR | CDS | DIS3 | CDS | IRF4 | CDS |
| CARD11 | CDS | DNM2 | Exon8/13/16/18/20 | JAK1 | CDS |
| CASP8 | Exon10 | DNMT3A | CDS | JAK2 | CDS |
| CBL | CDS | DNMT3B | CDS | JAK3 | CDS |
| CCND1 | CDS | EED | CDS | KDM6A | CDS |
| CCND2 | Exon4-5 | EGR1 | CDS | KIT | CDS |
| CCND3 | CDS | EP300 | CDS | KLF2 | Exon1-3 |
| CCR4 | CDS | ETNK1 | CDS | KMT2A | CDS |

| **Gene** | **Region** | **Gene** | **Region** | **Gene** | **Region** |
| --- | --- | --- | --- | --- | --- |
| KMT2D | CDS | PRKCB | CDS | TAL1 | Exon3 |
| KRAS | CDS | PRPS1 | CDS | TCF3 | Exon6/15/17 |
| MAP2K1 | Exon2-3 | PTEN | CDS | TERT | CDS |
| MAPK1 | CDS | PTPN11 | CDS | TET2 | CDS |
| MAX | CDS | RAD21 | CDS | TNFAIP3 | CDS |
| MED12 | CDS | RBBP6 | CDS | TNFRSF14 | Exon1-6 |
| MEF2B | Exon2-3 | RELN | CDS | TP53 | CDS |
| MPL | CDS | RHOA | Exon2-5 | TPMT | CDS |
| MYC | CDS | RPL10 | Exon5 | TRAF3 | CDS |
| MYD88 | CDS | RUNX1 | CDS | U2AF1 | CDS |
| NF1 | CDS | SETBP1 | CDS | USP7 | CDS |
| NOTCH1 | CDS | SETD2 | CDS | WHSC1 | CDS |
| NOTCH2 | CDS | SF1 | CDS | WT1 | CDS |
| NPM1 | CDS | SF3B1 | CDS | XPO1 | CDS |
| NRAS | CDS | SH2B3 | CDS | ZBTB7A | CDS |
| NT5C2 | Exon9-16 | SMC1A | CDS | ZMYM3 | CDS |
| PAX5 | CDS | SMC3 | CDS | ZRSR2 | CDS |
| PDGFRB | Exon18 | SPEN | Exon11 | PIGA | CDS |
| PHF6 | CDS | SRP72 | CDS | SRSF2 | CDS |
| PLCG1 | CDS | STAG2 | CDS | PPM1D | CDS |
| PLCG2 | CDS | STAT3 | CDS | STAT5B | Exon11/13-18 |
| PRDM1 | CDS | SUZ12 | CDS |  |  |
