# Supplementary material for: Mutational landscape of chronic myelomonocytic leukemia in Chinese patients
Source: Exp Hematol Oncol. 2022 May 24;11:32. doi: 10.1186/s40164-022-00284-z (PMC9128105; doi:10.1186/s40164-022-00284-z)
Supplement: Supplementary file 2 — Additional file 2: Table S2. Reference database website. [file 40164_2022_284_MOESM2_ESM.docx]

**Additional file 2: Table S2.** Reference database website.

| **Database** | **Website** |
| --- | --- |
| **Population**  dbSNP  ExAc  ESP6500  1000 Genomes | https://www.ncbi.nlm.nih.gov/snp/ |
|  | http://exac.broadinstitute.org |
|  | http://evs.gs.washington.edu/EVS/ |
|  | <http://phase3browser.1000genomes>. org/index.html |
| **Protein function prediction**  SIFT  PolyPhen2  MutationTaster  dbscSNV  **Somatic Mutations**  COSMIC | http://sift.jcvi.org |
|  | http://genetics.bwh.harvard.edu/pph2 |
|  | http://www.mutationtaster. org/ |
|  | https://sites.google.com/site/jpopgen/dbNSFP |
|  | <https://cancer.sanger.ac.uk/cosmic/> |
